# The goal of risk equalization in regulated competitive health insurance markets

**DOI:** 10.1007/s10198-022-01457-7

**Published:** 2022-03-29

**Authors:** Wynand van de Ven, Gerrit Hamstra, Richard van Kleef, Mieke Reuser, Piet Stam

**Affiliations:** 1 Erasmus University Rotterdam, Rotterdam, The Netherlands; 2 Equalis Strategy and Modeling, Utrecht, The Netherlands; 3 VU University Amsterdam, Amsterdam, The Netherlands

**Keywords:** Risk equalization, Efficiency, Level playing field, Risk selection, G18, I11, I13, I18

## Abstract

Different opinions exist about the goal of risk equalization in regulated competitive health insurance markets. There seems to be consensus that an element of the goal of risk equalization is ‘to remove the predictable over- and undercompensations of subgroups of insured’ or, equivalently, ‘to achieve a level playing field for each risk composition of an insurer’s portfolio’ or, equivalently, ‘to remove the incentives for risk selection’. However, the role of efficiency appears to be a major issue: should efficiency also be an element of the goal of risk equalization, or should it be a restriction to the goal, or should efficiency not be an element of the goal or a restriction to the goal? If efficiency plays a role, a comprehensive analysis of the total effect of risk equalization on efficiency needs to be done. An improvement of the performance of a risk equalization scheme has both negative and positive effects on efficiency. Negative effects include the reduction in efficiency via cost- or utilization-based risk adjusters. Positive effects result from leveling the playing field and reducing the incentives for risk selection, which increase efficiency as the outcome of a competitive market. In practice many regulators and policy makers take efficiency into consideration by looking at the negative effects, but hardly at the positive effects. The definition of the goal of risk equalization has consequences for the design and evaluation of risk equalization schemes and for the equalization payments. We describe relevant potential goals, tradeoffs and possible solutions.

## Introduction

The purpose of this paper is to discuss the question: “What is the goal of risk equalization in regulated competitive health insurance markets?” Many countries with a regulated competitive health insurance market have implemented a risk equalization scheme and are continuously improving it. A risk equalization scheme is a system of risk-adjusted equalization payments to and from (and within) the insurers that can be considered as risk-adjusted subsidies from low-risk enrollees to high-risk enrollees. After 30 years of gradual improvements of the risk equalization formula, the Dutch government thought that it was good enough and only needed regular maintenance. However, the health insurers opposed because several groups of insured, e.g., the chronically ill, were still undercompensated. This led to a discussion in the Netherlands about: what is the goal of risk equalization? Although there seems to be consensus that an element of the goal of risk equalization is ‘to remove the predictable over- and undercompensations of subgroups of insured’, the role of efficiency appears to be a major issue: should efficiency also be an element of the goal of risk equalization, or not? In this context, the concept of efficiency refers to the technical, allocative and dynamic efficiency of the provision of care and health insurance.

The relevance of having a clear goal of risk equalization is that it guides policy makers and researchers to the ‘right’ evaluation criteria for evaluating the extent to which the goal of risk equalization is achieved. It also makes clear whether new risk factors should be included in the risk equalization formula, or not. Despite intense debates on the goal of risk equalization in the Netherlands, no consensus was achieved. Because other countries are dealing with similar problems, the question “What is the goal of risk equalization?” has international relevance.

The paper is structured as follows: first, we sketch why and how, after 30 years of applying risk equalization, this question became relevant in the Netherlands. Second, we provide a theoretical framework for a good understanding of the rationale and the context of risk equalization. Third, we discuss several opinions about the goal of risk equalization, which are related to efficiency. Fourth, we discuss the complex relation between risk equalization and efficiency. Finally, we discuss our conclusions and the relevance for other countries.

## Risk equalization in the Netherlands

The risk equalization scheme in the Netherlands has been implemented in 1993 and gradually improved over time. Because of its poor performance in the early years most people agreed with any improvement that substantially reduced the incentives for risk selection and improved the level playing field for insurers. However, after 30 years of continuous improvements, it appears that the devil is in the details. Although the Dutch risk equalization scheme is now quite sophisticated and relatively good, it is still imperfect because several groups of insured are (substantially) over- or undercompensated [1]. And there is hardly any low-hanging fruit left for further improvements. Although there is consensus in the Netherlands that (1) risk equalization should preferably be prospective (i.e., based on expected spending rather than actual spending) and that (2) the undercompensation of chronically ill people should be avoided, the opinions about whether and how to remove the other over- and undercompensations diverge, resulting in intense discussions about controversial risk factors such as [2]^1^:

- regional differences (supply, prices, …),
- lifestyle,
- consumption propensity,
- health literacy,
- choice of voluntary deductible,
- migration background,
- yes/no seasonal worker,
- yes/no welfare recipient,
- yes/no defaulter,^2^
- yes/no homeless,
- switching behavior,
- yes/no giving birth in year of compensation,
- yes/no dying in year of compensation.

In 2020, the government initiated a project with the aim to find consensus among the insurers, researchers, and the government. To avoid subjective opinions about the goal and the design of risk equalization, the point of departure of this project was the question: “What is the goal of risk equalization based on the current legislation?”. However, despite intense discussions, no consensus was achieved [3–5].

## The rationale of risk equalization

This section provides a theoretical framework for a good understanding of the rationale and the context of risk equalization. First, we discuss the ‘equivalence principle’ of a competitive insurance market and why such a market results in accessibility and affordability problems. Second, we discuss the motives and effects of common regulatory interventions to make health insurance accessible and affordable, as well as the problems induced by that regulation. Third, we discuss the rationale of risk equalization, a tool for removing these regulation-induced problems.

### Equivalence of premium and expected costs per contract

Competitive markets for individual health insurance tend toward *equivalence* between the premium and an insurer’s expected costs for *each insurance contract*. The insurer’s expected costs of a health insurance contract are equal to the expected medical claims plus the loading fee, which covers the expected costs of matters such as the administration of contracts and claims, healthcare purchasing, building up solvency reserves and a compensation for risk-bearing. The equivalence principle implies that an insurer’s expected financial result (i.e., premium minus expected costs) tends to be equal for every insurance contract. That is, *ex ante every enrollee is equally ‘financially attractive’ for the insurer*. The raison d'être of the *equivalence principle* is that in a competitive insurance market insurers cannot compensate predictable losses on the contracts with the high risks by making predictable profits on the low risks, because competition minimizes predictable profits. Insurers can use (a combination of) the following three different tools to achieve equivalence of premiums and expected costs per contract [6]:

1. Premium differentiation per insurance product^3^: asking different premiums for the same insurance product by adjusting, for each insurance contract, the premium to the risk that the insured generates for the insurer.
2. Product differentiation: adjusting the insurance products (e.g., coverage, benefits design, panel of contracted providers) to attract different risk groups per product (i.e., risk segmentation) and charge premiums accordingly.^4^
3. Risk selection: adjusting the accepted risk to the stated premium of a given product, e.g., by refusing applicants or by excluding care for pre-existing medical conditions from coverage.

Even in health insurance markets where risk-rated premiums are allowed, insurers do not fully adjust their premiums to the underlying individual risk, e.g., because the necessary information may not be available or only at very high cost, or because insurers fear that this may harm their reputation, or because some groups of homogeneous risks are very small. In addition to premium differentiation, health insurers typically also use the other two tools (product differentiation and risk selection).^5^ By offering different insurance products, insurers can encourage self-selection, for example by offering a product with a high deductible to attract low-risk individuals. And by refusing high-risk applicants or by excluding treatments for pre-existing medical conditions from coverage, health insurers can select risks directly. Product differentiation and risk selection may be attractive for insurers because these strategies may be less expensive than refined premium differentiation and are likely to be less visible than charging extremely high premiums.

### Regulation to guarantee access to affordable health insurance coverage

Because of premium differentiation, product differentiation and risk selection, basic coverage in a free competitive health insurance market becomes unaffordable or inaccessible for many high-risk individuals. In many countries, this outcome is considered unacceptable and leads regulators to intervene. A direct way for the regulator (government) to guarantee affordability and accessibility of basic coverage is to forbid that health insurers use the above-mentioned three tools to achieve equivalence. That is, the regulator can implement:

1. a *ban on premium differentiation*, e.g., mandatory community rating (per product, if different versions of the basic cover are allowed),
2. a *standardized* basic-benefits package,
3. and an *acceptance duty* for such basic coverage (open enrollment).

In practice, in most countries with a competitive market for individual health insurance, the regulator has chosen for this type of regulation.

### Regulation-induced problems

The goal of this regulation is to enforce *implicit* cross-subsidies from the low risks to the high risks by forcing the insurers to use the predictable profits on the low risks to internally compensate for the predictable losses on the high risks. However, this regulation induces the problem that consumers differ substantially in their financial attractiveness for the insurers: high-risk people generate a predictable loss for the insurers, and low-risk people a predictable gain.^6^ Consequently, an insurer’s expected financial result depends on the risk composition of its portfolio. An insurer with an overrepresentation of high-risk insured must ask a higher premium than insurers with an overrepresentation of low-risk insured. This implies that there is *no level playing field for insurers* and that there are *incentives for subtle forms of risk selection*.^7^

#### No level playing field

In this context, a level playing field can be defined as a situation in which “an insurer’s expected financial result in year t is not dependent of the risk composition of his insurance portfolio in year t”. When such a level playing field exists for each potential risk composition of an insurer’s portfolio,^8^ two insurers who are identical in all aspects (including, for example, their insurance conditions, their contracting practices, their premium and their financial reserves) except the risk composition of their insurance portfolio, have an identical expected financial result.

An unlevel playing field is problematic for at least three reasons. First, the adversely selected insurers must ask a higher premium than their competitors. They may therefore lose market share and ultimately go bankrupt, even if they are efficient. Second, since premiums will not only reflect variation in ‘value’ but also the effect of risk selection, selection-driven premiums distort the consumers’ price/quality tradeoff. Third, it is hard for an insurer to set the premium for the next contract period such that the premium covers the expected costs, because prior to the next contract the insurer does not know how many unprofitable high-risk people he must accept during the open enrollment period. This may result in high loading fees as a compensation for bearing the risk or in the bankruptcy of adversely selected insurers.

In sum, an unlevel playing field may lead to the bankruptcy of adversely selected insurers, to a distortion of the consumers’ price/quality tradeoff and to higher loading fees.

#### Risk selection

With a ban on premium differentiation the low-risk insured are overpriced, and the high-risk insured are underpriced. Ideally, for each insurer the predictable losses on its high-risk enrollees should be compensated by the predictable profits on its low-risk enrollees. However, this ideal situation may not be achieved because of selection. *Selection* can be defined as “actions^9^ by consumers and insurers to exploit unpriced risk heterogeneity and break pooling arrangements” [7].^10^ Often the term selection is also used to refer to the outcome of these actions. Despite the open enrollment requirement there can be many forms of risk selection, for example, distorting the quality level of the offered insurance contracts (service-level distortion), providing the contracted doctors and hospitals with incentives for risk selection, selective advertising and marketing, selection via insurance agents, group contracts, or supplementary insurances.

Risk selection may be undesirable because of its adverse effects [9]. First, health insurers have a disincentive to respond to the preferences of high-risk consumers.^11^ The most worrisome form of selection is service-level distortion, e.g., by underprovision of services preferred by the high-risk insured and overprovision of services preferred by the low-risk insured [e.g., 11–13]. For this type of risk selection, it is not necessary that insurers know *which* individuals are high-risk or low-risk. It is sufficient for them to know *that* high-risk patients with disease X who have relatively strong preferences for good quality of treatment Y are undercompensated. Insurers may then skimp the quality of treatment Y. They may choose not to contract with providers who have the best reputations for treating the diseases of the undercompensated insured. This in turn can discourage physicians and hospitals from acquiring such a reputation. That would be an undesirable outcome of a competitive healthcare system.^12^ Even if all insurers are equally successful in this type of selection and therefore have the same risk composition of insured, this type of risk selection threatens the quality of care for the high-risk patients (see e.g., [14]).

Another possible outcome is that some insurers specialize in care for undercompensated high-risk patients and charge them a relatively high premium. In that case, the high-risk patients receive good care and good services only if they are able and willing to pay the high premium. The cross-subsidies as intended by the regulator may then not be fully achieved.

Second, another potential effect of risk selection is a reduction of efficiency. In case of large predictable profits resulting from selection, selection might be more profitable than improving efficiency in healthcare production. At least in the short run, when an insurer has limited resources available to invest in cost-reducing activities, it may prefer to invest in selection rather than in improving efficiency. Even if all insurers are equally successful in selection (and therefore no insurer has a selective risk composition of insured), their incentives for efficiency are reduced, at least in the short run. In addition, efficient insurers who do not engage in risk selection, may lose market share to inefficient risk-selecting insurers, resulting in a welfare loss to society.

Third, all forms of selection may result in market segmentation with the high-risk and low-risk insured choosing different health insurers or health insurance contracts with different (community-rated) premiums. Consequently, (1) the cross-subsidies as intended by the regulator may not be fully achieved, (2) premium differences do not only reflect differences in efficiency in healthcare production but also differences in risk composition, and (3) there is no level playing field for the insurers.

Finally, resources are wasted, since investments that are purely aimed at attracting low risks by risk segmentation or selection, produce no net benefits to society (zero-sum game among health insurers).

In sum, risk selection may lead to a reduction of the quality of certain types of care, a reduction of efficiency in healthcare production, and a reduction of the affordability of health insurance for the high risks.

### Risk equalization

Risk equalization is one of the tools to remove the regulation-induced problems listed in “Regulation-induced problems”.^13^ A *risk-equalization* scheme is a system of risk-adjusted equalization payments to and from (and within) the insurers that can be considered as *explicit* risk-adjusted subsidies from the low risks to the high risks. The equalization payments are based on risk characteristics of the insured (such as age, gender, and health status) that are used to predict the insured’s healthcare expenses. There are many ways of calculating the equalization payments^14^ and organizing these payments flows (see e.g., [15]). The regulator must decide which risk factors should be included in the risk equalization and which level of cross-subsidies is desired. For example, in the Netherlands regional characteristics are included as risk factors in the nation-wide risk equalization formula, while in Switzerland the risk equalization is done per canton. Consequently, in the Netherlands there are cross-subsidies among regions, while in Switzerland there are no transfers among the cantons.

Risk equalization with respect to only health expenses, as is currently the case in e.g., the Netherlands and Switzerland, is not sufficient to remove all regulation-induced problems mentioned in “Regulation-induced problems”. The reason is that the insurance premium contains, on top of the expected health expenses, a loading fee^15^ to cover e.g., administrative costs, the costs of building up financial reserves, and a compensation for bearing risk; and these costs are higher for high-risk than for low-risk enrollees.^16^ So without an adequate equalization for these expenses, there is still no level playing field and there are still incentives for risk selection. For a discussion how and to what extent the loading fee should be equalized, see [16].

After risk equalization, different insurance products may have on average different expected costs per enrollee. As far as these differences in expected costs are due to risk selection, they should be equalized without compensating for the managed care effects and the difference in out-of-pocket expenses. As far as these differences in expected costs are the result of differences in managed care or differences in out-of-pocket payments, they should not be equalized and can be reflected in the community-rated premiums of these insurance products.^17^ Therefore, the characteristics of the insurance products (e.g., cost-sharing arrangements, the panel of contracted providers, and the managed care clauses) need not to be equalized.^18^

If different specifications of the equalization scheme achieve the same equalizing effect, the regulator may choose, for example, the specification that is easiest to implement, or that (on balance; see “Positive and negative effects on efficiency”) results in the most efficiency, or that removes the most severe regulation-induced problems (see “Priority setting and constrained regression”).^19^

#### Risk sharing

It is an unanswered empirical question to what extent risk equalization alone can be sufficient to remove all regulation-induced problems mentioned in “Regulation-induced problems”. Most likely risk equalization can include more risk factors than the risk factors that insurers in a free market use in practice for premium differentiation. As explained in “Equivalence of premium and expected costs per contract”, there are several reasons why insurers do not fully adjust their premiums to the underlying individual risk. In addition, the regulator may be able to use more risk factors than the insurers can use if the regulator has access to information about risk factors that insurers do not have, as is the case, e.g., in the Netherlands. And risk groups that may be too small for an individual insurer to be used for premium differentiation, may be sufficiently large for the regulator to use in risk equalization. However, no risk equalization scheme currently in place fully compensates the insurers for unpriced risk heterogeneity.^20^ Consequently, additional tools are necessary to remove the in “Regulation-induced problems” mentioned regulation-induced problems. A straightforward tool is additional cost-based compensations, e.g., for the residual losses, given risk equalization, of applicants whom insurers in a free market would reject or only accept under certain conditions. We refer to these cost-based compensations as retrospective *risk sharing*.^21^ Risk sharing implies that insurers retrospectively receive certain payments based on their actual expenses. These payments are paid out of a fund that is filled with mandatory contributions. Several forms of risk-sharing can mimic the selective acceptance policy of insurers in a free market (see e.g., [15, 18, 19]. For example, each insurer could be allowed to ex-ante designate a specified percentage of his enrollees (for example, 1 or 4 percent) for whom the costs will be compensated. This ‘risk sharing for high-risks’ simulates the rejection of applicants that could occur in a free competitive market. Another form of risk sharing is that insurers can be fully or partly compensated for an individual’s expenses above a certain annual threshold. Such cost-based subsidies can substantially reduce the insurers’ costs for insured with extremely high healthcare expenses. Consequently, the undercompensations for these enrollees and the incentives for risk selection against them are reduced. However, there is a tradeoff because risk sharing not only reduces the incentives for risk selection, but also reduces the insurers’ incentives to control cost. This complicated tradeoff will be discussed in “Risk equalization and efficiency: a complex relation”.

## Opinions about the goal of risk equalization

### The Netherlands

Although risk equalization is generally considered as an important tool to remove the regulation-induced problems listed in “Regulation-induced problems”, opinions about the precise goal of risk equalization differ. Based on recent policy documents, an overview will be given of the several opinions about the goal of risk equalization that have been put forward in the discussion in the Netherlands, and their rationale. In the Netherlands, there is a competitive market for individual health insurance, a mandate for everyone to buy a standard basic health insurance coverage, an annual open enrollment, mandatory community rating per health insurance contract,^22^ and a risk equalization system. Each insurer can offer different versions of the standard basic cover, e.g., with different panels of contracted providers, or with different conditions for receiving certain types of care. Although a different interpretation of the current legislation plays a major role in the discussion, in this paper we focus only on economic and policy arguments, and we waive legal arguments regarding Dutch and European Union legislation that came up in the discussion.^23^ The following formulations of the “goal of risk equalization” have been put forward.

**Goal-A.** *“to achieve that ‘each applicant whom an insurer must accept’ forms an equal insurance risk for the insurer”.*

This definition has been directly derived from the Dutch legislation.^24^ It gives rise to the question: *What is an ‘equal insurance risk’?* We could interpret it as: each applicant whom the insurer must accept, should ex-ante be equally ‘financially attractive’ for the insurer, as far as health insurance is concerned.^25^ This implies that goal-A is equivalent to ‘*remove the predictable over- and undercompensations of subgroups of insured’* or, equivalently, “*achieve a level playing field for each risk composition of an insurer’s portfolio”* or, equivalently, “*remove the incentives for risk selection*”.^26^ Achieving goal-A requires that all risk factors that insurers are not allowed to use for premium differentiation should be included in the risk equalization.^27^ This holds for risk factors such as age-sex, health status, socio-economic factors (income, education), characteristics of providers (e.g., practice style and price), and characteristics of the region (e.g., whether there is an oversupply of providers and facilities). In addition, it is stated in the Dutch legislation that to the extent that the goal of risk equalization is not achieved, risk equalization should be complemented with risk sharing.^28^

**Goal-B.** *“to achieve that ‘each applicant whom an insurer must accept’ forms an equal insurance risk for the insurer, under the restriction that the ‘precondition that the incentives for efficiency are not too strongly reduced’ is of equal importance as the achievement of the goal”*[4, p. 1–2], [3, p. 9–10].

Goal-B is like goal-A, but with a restriction. The rationale of this restriction is that improving efficiency is one of the goals of the healthcare system and therefore the risk equalization should guarantee the incentives for efficiency as much as possible. For example, those risk factors that can immediately be influenced by insurers, should not be included in the risk equalization. This restriction raises some questions. What is meant by ‘*not too strongly’*? What means *immediate* influence? Which criteria to use when making the tradeoff implied by the restriction?

**Goal-C.** *“to achieve a level playing field, to avoid risk selection, and to increase efficiency.”*^29^ Goal-C is similar to goal-A, but extended with the goal to also achieve efficiency. Goal-C and goal-B are very close, and have the same rationale.

**Goal-D.** *“to pursuit that ‘each applicant whom an insurer must accept’ forms an equal insurance risk for the insurer as far as objective health is concerned; and to sufficiently achieve that ‘each applicant whom an insurer must accept’ forms an equal insurance risk for the insurer.”*[4, p. 5–7].

The rationale of goal-D is that there should be no equalization, for example, for risk factors that the insurer can influence (either in the short or the long term)^30^ and no equalization for characteristics of the providers. Goal-D raises the following questions. What exactly is *objective* health? What exactly means ‘*sufficient’*? How can it be evaluated whether goal-D is achieved?

With Goal-A, efficiency should not be an element of the goal, but only one of the potential selection criteria for choosing among different equalization schemes. With goal-B and goal-C, efficiency should be a restriction to or an element of the goal of risk equalization, respectively. Goal-D is similar to goal-A, but there is only a best-efforts obligation rather than a result obligation, and with the restriction that only *objective* health is allowed to be a risk factor in the risk equalization. Another difference with goal-A is that in case of a *sufficient* equal insurance risk there is no need for further risk equalization or risk sharing to achieve an equal insurance risk.

The majority of the Dutch experts on risk equalization are in favor of goal-B [4].

### International experts

At a conference^31^ of the Risk Adjustment Network (RAN^32^) we presented the above-mentioned goals of the risk equalization. We asked the participants—25 international experts on risk equalization—what they thought is the goal of risk equalization. It turned out that their answers were quite diverge. Interpreting their answers in terms of the above four specified goals, each of the goals-A, -B and -C were roughly equally chosen.

At a conference on risk equalization in Germany^33^ we also presented the above-mentioned goals of risk equalization and asked the 50 German experts on risk equalization what they thought is the goal of risk equalization. It turned out that (again) each of the goals-A, -B and -C were roughly equally chosen.

So, there are indications that also among international experts differing opinions exist about the goal of risk equalization.

### Conclusion

Despite the diverging opinions on the goal of risk equalization, there seems to be consensus that an element of the goal of risk equalization is ‘to remove the predictable over- and undercompensations of subgroups of insured’ or, equivalently, ‘to achieve a level playing field for each risk composition of an insurer’s portfolio’ or, equivalently, ‘to remove the incentives for risk selection’. However, the role of efficiency appears to be a major issue: should efficiency also be an element of the goal of risk equalization (goal-C), or should it be a restriction to the goal (goal-B), or should efficiency not be an element of the goal or a restriction to the goal, and therefore only be one of the potential selection criteria for choosing among different equalization schemes (goal-A)?

From the three above indicated groups of experts on risk equalization a minority is in favor of goal-A and a majority is in favor of either goal-B or goal-C. In other words: according to a minority the goal of risk equalization is to remove the predictable over- and undercompensations of subgroups of insured, and a majority gives a roughly equal weight to ‘removing the over- and undercompensations of subgroups’ and increasing or guaranteeing efficiency.

These different goals have serious consequences for the design of the risk equalization system. For example, whether the risk factors that are mentioned in “Risk equalization in the Netherlands” should be included in the risk equalization formula, depends on the elected goal. It has also consequences for the criteria to evaluate whether and to what extent the goal of risk equalization is (not) achieved.

## Risk equalization and efficiency: a complex relation

### Positive and negative effects on efficiency

In general, efficiency is assumed to be the outcome of a competitive market. For competitive healthcare markets several preconditions must be fulfilled to achieve efficiency and affordability [21]. One of these preconditions is a system of cross-subsidies without incentives for risk selection. If this precondition is not fulfilled, the regulation-induced problems *unlevel playing field for insurers* and *incentives for risk selection* occur, which reduce the incentives for efficiency (and have the other negative effects as discussed in “Regulation-induced problems”). So, improving the performance of a risk equalization scheme increases efficiency as the outcome of a competitive market by leveling the playing field and reducing the incentives for risk selection. Efficiency will be increased because, for example: (1) insurers will be less focused on selection activities and more focused on efficiency, (2) quality skimping will be reduced which, keeping costs equal, improves efficiency, (3) the premium competition on the health insurance market will increase the insurers’ incentives for efficiency because the premiums are less selection-driven and more efficiency-driven, (4) selection-driven product differentiation will be reduced which increases transparency on the health insurance market and thereby facilitates a value-for-money consumer choice of health insurance, (5) the probability of bankruptcies of (efficient) insurers resulting from adverse selection will be reduced, and (6) wasteful resources spent on selection activities will be reduced.

However, in practice risk equalization comes with a price. Ideally the risk adjusters used in the risk equalization should be valid, reliable, and non-manipulable, and the required data must be available at socially acceptable costs. Because of these conditions, it is likely that the goal of risk equalization can only be achieved at a high price, such as high costs to collect the required data or including risk factors that decrease the incentives for efficiency. For example, state-of-the-art ‘risk equalization’ schemes have diagnoses/cost-based risk adjusters which can reduce the insurers’ incentives for efficiency and lead to incentives for gaming. Equalization payments based on prior diagnoses might also reduce incentives for prevention (for example, by discouraging lifestyle interventions for -potential- diabetes patients) and stimulate further and more expensive treatments.

So, the relation between risk equalization and efficiency is complex.^34^ On the one hand, an improvement of the performance of risk equalization schemes improves efficiency via leveling the playing field and reducing the incentives for risk selection, and thereby increasing efficiency as the outcome of a competitive market. But on the other hand, an extension of the risk equalization with additional risk adjusters may have negative effects on the incentives for efficiency through increased incentives for gaming and/or reduced incentives for cost efficiency and prevention. To know the overall net effect on efficiency, a comprehensive analysis needs to be done of both the negative and positive effects.^35^

For goal-B and goal-C it is necessary to perform such a comprehensive analysis. However, in practice many regulators and policy makers consider the negative effects, and not the positive effects. For example, according to the Swiss legislation an improvement of the risk equalization should not reduce the insurers’ efficiency, which in practice is interpreted as a ban on risk factors such as prior expenses [23]. Also, for decades Dutch policy makers and insurers hardly considered positive effects on efficiency while improving the risk equalization by adding new predictive risk factors to it.

### The incentives of the insured for efficiency

It is an intriguing question why policy makers worry so much about potential risk factors in the risk equalization formula that potentially reduce an insurer’s incentive for efficiency. In free competitive insurance markets insurers often use such risk factors, and nevertheless such competitive markets are generally considered to be efficient. So, it is an intriguing question why risk factors that potentially reduce an insurer’s incentive for efficiency, are a (potential) problem in risk equalization and not in a free market with risk rating.

A possible explanation is that in a free competitive market the insured have substantial incentives for efficiency.^36^ In such a market the high risks pay a risk-adjusted high premium. If premiums depend on e.g., prior year’s expenses, diagnoses or prescription drugs, the insured have a strong incentive for efficiency, e.g., by reducing moral hazard and supply-induced demand, because that would substantially reduce their future premium. Risk-adjusted premiums may also stimulate the insured to engage in prevention and to reduce their health risk because that will reduce their future premium. For example, a diabetic patient who changes her lifestyle such that she is no longer a diabetic patient, will then pay a substantially lower premium. However, the premium regulation, e.g., mandatory community rating per product, results in a complete removal of these incentives for efficiency. So, if policy makers worry about incentives for efficiency, they may consider fixing this regulation-induced problem by increasing the incentives of the insured for efficiency via effective forms of cost-sharing (e.g., [24]), or by allowing the insurers to risk rate the consumer’s out-of-pocket premium within a certain bandwidth (see “Premium b and width”).

### Priority setting and constrained regression

In the case of goal-B, -C and –D, the result will be over- and undercompensations of subgroups (i.e., an unlevel playing field and incentives for risk selection) because of the tradeoff with efficiency.^37^ When making this tradeoff it is recommended to give priority to avoiding the potential effects of an unlevel playing field and incentives for risk selection (as mentioned in “Regulation-induced problems”) that are socially the most undesirable. A high priority could be given to the prevention of selection actions that reduce the quality of care used by undercompensated insured, e.g., the chronically ill and people with a mental health problem.^38^ Ideally, we would like to see the insurers advertise for each chronic disease and mental health problem with: “Come with us, we have contracted the best providers specialized in your disease!”. Unfortunately, so far, we have not seen such advertisements in any of the countries with a regulated competitive health insurance market. This is a high price for society that cannot be easily compensated by an increase in efficiency.

The prevention of a ‘reduction of the quality of care for undercompensated chronically ill people via service-level distortion’ requires the elimination of their undercompensation. The natural approach to do so is enriching the risk equalization scheme with risk adjusters that indicate membership of the relevant groups. For some groups, however, appropriate risk adjusters cannot (yet) be used, e.g., because the relevant information is not (yet) available for all insured or because the use of this risk adjuster would result in gaming possibilities for insurers or other perverse incentives. For these situations, an alternative approach is to reduce the under- or overcompensations by constraining the estimated coefficients of the risk-equalization scheme such that the under- or overcompensation of the groups of interest equal a fixed amount. It is shown that, compared to ordinary least-squares, such *constrained regressions* can reduce the undercompensations of some groups, e.g., the chronically ill, but increase undercompensation of others [27]. Empirical findings have shown that the benefits of introducing constraints to eliminate the undercompensations for the chronically ill can be worth the costs in terms of undercompensations for others in the Dutch insured population. Therefore, constrained regression is a promising tool for fulfilling the priorities set by the regulator.

### Premium bandwidth

An effective tool for the regulator to level the playing field for insurers, to reduce risk selection, *and* to increase efficiency is to allow insurers to risk rate the consumer’s out-of-pocket premium within a certain bandwidth. Consequently, much additional information about the applicant’s risk that the insurers have, given the risk equalization, would be focused on premium differentiation rather than on risk selection. In this way the goal of risk equalization is better achieved, not only without any reduction of efficiency, but with increased efficiency as the outcome of the competitive market and with an increase of the incentives of the insured for efficiency (see “The incentives of the insured for efficiency”). Most likely, given a sophisticated risk-equalization system such as in the Netherlands, a premium bandwidth of a few hundred euros is sufficient to remove most of the remaining under- and overcompensations [1]. Such a bandwidth increases the premium for (most likely) the high-risk individuals. Because this premium bandwidth is a small fraction of what the premium bandwidth would be in a free competitive insurance market without risk equalization,^39^ leveling the playing field, reducing risk selection *and* increasing efficiency require a relatively small tradeoff with affordability (solidarity). Low-income high-risk individuals could receive a premium-related subsidy. If insurers are required to identify any risk factors they use for premium differentiation, the regulator could try to include these risk factors in the risk-equalization formula in subsequent years. Potentially, such market-driven improvements of the risk equalization mechanism may be more effective and more workable than research-driven improvements, but they confront the regulator with a (temporary) tradeoff between affordability and the negative effects of an unlevel playing field and risk selection (as mentioned in “Regulation-induced problems”), including efficiency.

## Conclusion and discussion

Different opinions exist about the goal of risk equalization, not only among health insurers, researchers, and government in the Netherlands, but also among international experts. There seems to be consensus that an element of the goal of risk equalization is ‘to remove the predictable over- and undercompensations of subgroups of insured’ or, equivalently, ‘to achieve a level playing field for each risk composition of an insurer’s portfolio’ or, equivalently, ‘to remove the incentives for risk selection’. However, several people find the focus on only these elements too strict because they do not take *efficiency* explicitly into consideration, while improving efficiency is one of the goals of competitive healthcare markets. So, a major issue is: should efficiency also be an element of the goal of risk equalization (goal-C), or should it be a restriction to the goal (goal-B), or should efficiency not be an element of the goal or a restriction to the goal, and therefore only be one of the potential selection criteria for choosing among different equalization schemes (goal-A)?

In this paper we described the opinion of three groups of experts on risk equalization. According to a minority of them the goal of risk equalization is only to remove the predictable over- and undercompensations of subgroups of insured, and a majority gives a roughly equal weight to ‘removing the predictable over- and undercompensations of subgroups’ and ‘efficiency’ when formulating the goal of risk equalization.

However, the relation between risk equalization and efficiency is complex. On the one hand, an extension of the risk equalization may have negative effects on the incentives for efficiency through increased incentives for gaming and/or reduced incentives for cost efficiency and prevention. But on the other hand, an improvement of the performance of a risk equalization scheme improves efficiency by leveling the playing field and reducing the incentives for risk selection, and thereby increasing efficiency as the outcome of a competitive market. Efficiency will be increased because, for example: (1) insurers will be less focused on selection activities and more focused on efficiency, (2) quality skimping will be reduced which, keeping costs equal, improves efficiency, (3) the premium competition on the health insurance market will increase the insurers’ incentives for efficiency because the premiums are less selection-driven and more efficiency-driven, (4) selection-driven product differentiation will be reduced which increases transparency on the health insurance market and thereby facilitates a value-for-money consumer choice of health insurance, (5) the probability of bankruptcies of (efficient) insurers resulting from adverse selection will be reduced, and (6) wasteful resources spent on selection activities will be reduced. When it comes to the evaluation of a potentially new risk adjuster, a comprehensive analysis needs to be done not only of the negative effects of that new risk adjuster on efficiency (e.g., in terms of gaming or upcoding), but also of the positive effects on efficiency as mentioned above. For goal-B and goal-C it is necessary to perform such a comprehensive analysis. However, in practice many regulators and policy makers who aim at improving the risk equalization by adding new predictive risk factors to it, take efficiency into consideration by looking at the negative effects on efficiency, and hardly at the positive effects on efficiency.

In the case of goal-B and goal-C, the result will be an unlevel playing field and incentives for risk selection because of the tradeoff with efficiency. When making this tradeoff it is recommended to give priority to avoiding the potential effects of an unlevel playing field and incentives for risk selection (as mentioned in “Regulation-induced problems”) that are socially the most undesirable. A high priority could be given to the prevention of selection actions that reduce the quality of care used by undercompensated insured, e.g., the chronically ill. Note that, compared to ordinary least-squares, *constrained regression* is a promising tool for fulfilling this priority.

In the case of goal-A society most likely is not willing to achieve the goal (i.e., remove the predictable over- and undercompensations of subgroups of insured) at any cost, and will therefore make a tradeoff between the advantages and disadvantages of coming closer to the goal. The advantages are a further reduction or even elimination of the in “Regulation-induced problems” mentioned regulation-induced problems, while the disadvantages can be a reduction of the incentives for efficiency, high costs of collecting the relevant data, and creating opportunities and incentives for manipulation of the data by the insurers. Most likely, politicians will give different weights to the different regulation-induced problems that may occur. For example, they could give a very high weight to avoiding service level distortion that may result in lower quality of care for the chronically ill people, and a lower weight to avoiding that those who choose a voluntary deductible have a premium rebate that, due to risk selection, is, say, one hundred euro per year higher than it would be without risk selection.

An effective tool for the regulator to level the playing field for insurers, to reduce risk selection, *and* to increase efficiency is to allow insurers to risk rate the out-of-pocket premium of the insured within a certain bandwidth. Given a sophisticated risk-equalization system this premium bandwidth is a small fraction of what the premium bandwidth would be in a free competitive insurance market without risk equalization. However, allowing risk-rated premiums comes at a price, namely a reduction of the affordability of health insurance, especially for those with relatively high health care spending. When a relatively high weight is given to affordability in the tradeoff with leveling the playing field, reducing risk selection *and* increasing efficiency, a premium bandwidth could be combined with premium-related subsidies for low-income high-risk individuals.

The different formulations of the goal of risk equalization have serious consequences for the design of the risk equalization scheme and for the level of compensations via the risk equalization. For example, should the risk factors that are mentioned in “Risk equalization in the Netherlands”, be included in the risk equalization formula, or not? It has also consequences for the criteria to evaluate to what extent the goal of risk equalization is achieved. For example, should researchers exclusively focus on the extent to which risk equalization mitigates predictable profits and losses? Or should they also quantify the (positive and negative) effects of risk equalization on incentives for efficiency? Academics can advise and indicate the tradeoffs and the consequences of several formulations of the goal of risk equalization, but at the end of the day it is the regulator who should clearly define the goal in a way that cannot be misinterpreted.

The discussion on the goal of risk equalization in the Netherlands became more intensive after 30 years of continuous improvements of the risk-equalization system, and when there was no low-hanging fruit left to further improve the quite sophisticated and relatively good risk equalization system. It can be expected that other countries with a regulated competitive health insurance market will face (at some point) a similar situation and intensify the discussion on the goal of risk equalization. This paper may help these countries to come to a consensus on the goal of risk equalization.

## Footnotes

1 WOR means Werkgroep Ontwikkeling Risicoverevening, i.e., “Working group Development Risk Equalization”. Participants of this Dutch working group, which meets about 8 times per year, are civil servants, representatives of insurers and researchers. The Ministry of Health is the chair and the secretary of the working group.

2 In this context, a defaulter is an insured who fails to fulfill his contractual duty of paying the health insurance premium.

3 An insurance product implies that the same insurance conditions apply to all enrollees who have the same insurance product, apart from the premium. For example, insurance contracts with different deductibles and otherwise the same insurance cover are considered to be different insurance products. The premium discount for a deductible is not regarded as premium differentiation per product, but as a premium discount compared to a product without deductible and with the same insurance conditions.

4 In the case of voluntary insurance an additional motive for product differentiation (e.g., high and low deductibles) is to offer low-risk people a contract that is more attractive for them than being uninsured.

5 Voluntary traditional (commercial) reinsurance is not a tool to achieve equivalence of premiums and expected costs per contract. Insurers can buy reinsurance to (1) increase their financial capacity to underwrite coverage, or (2) to reduce their legally required solvency reserves, or (3) to protect themselves against extremely high expenses and thereby reduce their risk of ruin. Reinsurance requires a risk-adjusted reinsurance premium, based upon the characteristics of the distribution of the expected medial claims. The expected costs of reinsurance have been considered by the insurer when setting the premium of the contract with the insured. Note that a mandatory reinsurance program with regulated reinsurance premiums may be a tool to achieve equivalence, we call this ‘risk sharing’ (see “Risk sharing”).

6 Another problem is a substantial reduction of the incentive of the insured for efficiency. See “The incentives of the insured for efficiency”.

7 Subtle forms of risk selection are all forms of risk selection except the forbidden forms of risk selection (i.e., refusing new applicants and excluding care for pre-existing medical conditions from coverage).

8 A level playing field must exist for each potential risk composition of an insurer’s portfolio and not only for the current risk compositions of the insurers’ portfolios, because the risk compositions of the insurers’ portfolios may change after the next open enrollment period.

9 Not including premium differentiation per health insurance contract.

10 As explained by Van Kleef et al. [8], the concept of “unpriced risk heterogeneity” (URH) from the viewpoint of insurers differs from that of the viewpoint of consumers. For insurers, URH relates to variation in gaps between the expected costs and revenues of insurance contracts. For consumers, URH relates to variation in gaps between expected costs and premiums of insurance contracts (net of subsidies). This distinction is relevant for this paper since risk equalization corrects for URH from the perspective of insurers, but not for URH from the perspective of consumers. Even in markets with sophisticated risk equalization, selection incentives for consumers remain to exist (e.g., when it comes to the decision whether to buy insurance and the decision which insurance plan to buy). These selection incentives for consumers cannot be corrected by risk equalization because risk equalization payments to insurers do not correct for variation in gaps between expected costs and premiums *from the perspective of consumers*. Instead, other tools are necessary such as an insurance mandate, premium differentiation, and external subsidies. This paper focusses on URH (and selection incentives) from the perspective of insurers.

11 For example, health insurers with a good reputation for chronic care would attract many unprofitable patients and would be the victim of their own success. Beaulieu et al. [10] discuss the case of a Health Maintenance Organization with a successful Diabetes Management Program, resulting in improved health outcomes and lower costs that eventually lost money because the program attracted a disproportionate share of diabetics.

12 The effect of service-level distortion may be more serious if an individual insurer is the purchaser of care on behalf of its enrollees (as e.g., in the Netherlands and the USA) than if all insurers collectively negotiate and collectively contract with all providers of healthcare (as e.g., in Germany and Switzerland).

13 Another tool for achieving a level playing field for insurers is, e.g., effective competition policy that prevents cartels and the abuse of dominant positions. Other tools for the prevention of risk selection are, e.g., to forbid (some) actions by insurers and consumers that result in risk selection, e.g., forbid the tying in sale of basic cover and supplementary insurance, forbid voluntary deductibles, and forbid a narrow panel of contracted providers. However, ‘taking away selection instruments’ (e.g., forbidding selective contracting) reduces possibilities for insurers to improve efficiency of care. In addition, the regulator can forbid only a restricted number of specific actions, while in practice there are countless subtle selection actions possible.

14 For example, the equalization payment per insured could be equal to the insured’s risk-adjusted predicted expenses minus a fixed amount (e.g., the average expenses per person). If the equalization payment is negative, the insurer must pay it to the equalization fund.

15 Depending on the characteristics of the health insurance market, the share of the loading fee is about 3 to 20 percent of total premium payments.

16 For example, administrative costs related to checking bills, fraud prevention, administrative contacts (phone, visits, email, etc.), costs for handling defaulters, purchasing healthcare, contracting healthcare providers, quality improvement, utilization management and the coordination of care are higher for an insurer with a high-risk population than one with a low-risk population. The level of risk is not only determined by the expected medical claims, but also by other characteristics of the distribution function of medical claims. For example, keeping expected medical claims constant, the costs of ‘bearing risk’ are an increasing function of the variance of the expected medical claims.

17 If insurers offer different insurance products with different premium levels, the regulator should decide about the ‘acceptable costs’ to be equalized [15], i.e., which costs are acceptable to be equalized? For example, should that be the costs of the insurance product with a narrow panel of contracted providers, or the costs of the product with a free choice of provider? Should it be the costs of the product with a high voluntary deductible, or the product without a voluntary deductible? In the Netherlands the regulator has (implicitly) decided that the acceptable costs are the weighted average costs of all insurance products that are offered in the market. Under the Affordable Care Act in the United States the regulator has made another choice. The health insurance products are categorized into four tiers based on how covered medical costs are shared between the insurer and the insured: Bronze, Silver, Gold and Platinum, with, respectively, 60%, 70%, 80% and 90% of medical costs covered by the insurer. For each of these metal tiers there is a separate risk equalization scheme, which implies that there are no cross-subsidies among the different metal tiers. In other words, the selection that is caused by the consumer’s choice of metal tier, is not reflected in the risk-equalization payments (but it is reflected in the premium of the insured). The level of ‘acceptable costs’ is also the appropriate level of costs to be used for evaluating the extent to which the goal of risk equalization is (not) achieved.

18 For a discussion of risk equalization and characteristics of the insurance-product (e.g., cost-sharing arrangements, the panel of contracted providers, and the managed care clauses) see [17]. A discussion of the case that there are interaction terms between the risk factors in the risk equalization and the characteristics of the insurance-product is beyond the scope of this paper.

19 The (perceived) seriousness of these regulation-induced problems may vary from country to country.

20 The feasibility of improving risk equalization may change over time by the availability of e.g., new data, new statistical methods, new insights, or faster computers.

21 We use the term ‘risk sharing’ and not the term reinsurance that (since a few years) the regulator, policy makers and academics in the USA often use. An essential difference between traditional reinsurance and risk sharing is that for reinsurance an insurer must pay a risk-adjusted premium to the reinsurer. Consequently, traditional reinsurance does not reduce the insurer’s predictable losses on high-risk individuals. It may increase them because of the loading fee included in the reinsurance premium. Therefore, traditional reinsurance cannot be a tool to reduce the insurer’s incentives for selection. (See also, mutatis mutandis, the argument given in footnote 5.) Risk sharing could be described as a “mandatory reinsurance program with regulated reinsurance premiums” as distinct from voluntary reinsurance with risk adjusted reinsurance premiums [15, p. 816]. In this context cost-sharing would be a better term than risk-sharing, but cost-sharing is a term that already for a long time is used to indicate the consumer’s out-of-pocket expenses (coinsurance, copayment, or deductible), i.e., sharing the costs between the insured and the insurer.

22 Without premium regulation the goal of risk equalization would be to make health insurance affordable for the high risks [15].

23 Optimal policy measures should not be restricted by obstructive legislation, but legislation should (be changed to) support optimal policy measures.

24 Explanatory Memorandum to the Health Insurance Act, Tweede Kamer, 2003–2004, 29,783, nr. 3, p. 49.

25 That is, we focus only on health insurance and do not take into account that some applicants may be more or less attractive in relation to characteristics of the insurer (e.g., the level of solvency reserves or the number of insured) or other insurance products of an insurer. We also do not consider that an insurer prefers certain groups of applicants to other groups because of the insurer’s area of specialization (e.g., specialized in care for certain groups of patients).

26 It can be easily seen that these four formulations are equivalent. For example, each applicant is ex-ante equally financially attractive for the insurer if and only if there are no predictable over- and undercompensations of subgroups of insured. And there is a level playing field for each risk composition of an insurer’s portfolio if and only if there are no incentives for risk selection. And there are no over- and undercompensations of subgroups of insured if and only if there are no incentives for risk selection.

27 In most countries the use of all risk factors is forbidden, and then there should be a full equalization for these risk factors. Sometimes a premium bandwidth is allowed. In that case the risk equalization should not compensate for the allowed premium differentiation. For example, under the Affordable Care Act in the United States health insurance premium rates can be adjusted for age (with a maximum pricing differential across age groups of 3:1), family size, geographical region, and smoking status (with a maximum pricing differential of 1.5:1) See e.g., [20].

28 Explanatory Memorandum to the Health Insurance Act, Tweede Kamer, 2003–2004, 29,783, nr. 3, p. 50–51.

29 Description of the risk adjustment system of the Health Insurance Act, publication by the Ministry of Health, Welfare and Sport, March 2016, p. 3–5.

30 An insurer is considered not to be able to influence objective health.

31 In Weggis, Switserland, 2–3 September 2021.

32 See http://www.riskadjustment.net.

33 The conference “3. RSA Fachkongress”, organized by WIG2, 20–21 October 2021, Leipzig RSA-Fachkongress 2021—WIG2—Wissenschaftliches Institut für Gesundheitsökonomie und Gesundheitssystemforschung.

34 The relation between risk equalization and efficiency can become even more complex if risk equalization does not apply to all risk factors and insurers are free to risk rate their premium for the remaining set of risk factors. Schokkaert et al. [22] have shown that if the healthcare expenditure function is not additively separable in these two sets of risk factors, a conflict (tradeoff) between efficiency and risk selection is unavoidable, even in the case of perfect information about the medical expenditure function. A discussion of this complexity is outside the scope of this paper.

35 The same holds for the introduction or an extension of risk sharing.

36 Another possible explanation might be that risk equalization increases the rent seeking activities of the insurer (sometimes addressed as ‘gaming’). However, it is an unanswered empirical question whether rent seeking activities in the case of risk equalization are larger than with free premium differentiation and without risk equalization.

37 This is consistent with the ‘Tinbergen [25] rule’ that each policy objective should be addressed by a single tool (or, alternatively, for each policy objective there should be at least one tool).

38 This recommended priority holds in particular for countries where an individual insurer is the purchaser of care on behalf of its enrollees. This is the situation in most countries with a competitive health insurance market and risk equalization. Two exceptions are Germany and Switzerland, where there are collective negotiations and collective contracts between all insurers and all providers of healthcare (per state). In Germany a high priority is given to the prevention of manipulation of risk factors by the insurers (see [26]).

39 Because of the huge variation in predictable health expenses among individuals, premium differentiation in a free competitive health insurance market could result in a premium bandwidth with the highest premium being more than a 1000-fold of the lowest.
